# Concomitant systemic inflammation and cellular immunosuppression in patients with Cushing's syndrome

**DOI:** 10.1002/ctm2.1314

**Published:** 2023-07-13

**Authors:** Pepijn van Houten, Chunying Peng, Martin Jaeger, Antonius E. van Herwaarden, Mihai G. Netea, Annenienke C. van de Ven, Romana T. Netea‐Maier

**Affiliations:** ^1^ Department of Internal Medicine Division of Endocrinology Radboud University Medical Center Nijmegen The Netherlands; ^2^ Department of Laboratory Medicine Radboud University Medical Center Nijmegen The Netherlands; ^3^ Department of Internal Medicine and Radboud Center for Infectious Diseases Radboud University Medical Center Nijmegen The Netherlands

Dear Editor,

Cushing's syndrome (CS) is characterised by prolonged endogenous hypercortisolism and is associated with an increased incidence of cardiovascular complications and a higher susceptibility to infections.^1^ This study shows that patients with active CS present concomitant cellular immunosuppression and increased circulating concentrations of proinflammatory cytokines, reflecting systemic inflammation, which are likely to at least partially underlie the cardiovascular and infectious complications.

Increasing evidence shows that innate immunity is involved in the pathogenesis of these complications. Recently, our group described that innate immune cells can undergo functional reprogramming characterised by hyperresponsiveness to subsequent unspecific stimuli, a phenomenon referred to as *trained immunity*.^2^ Other studies have shown that trained immunity is involved in the pathogenesis of cardiovascular diseases.^3^ Recurrent exposure to stimuli can, on the other hand, induce immune exhaustion, leading to a higher susceptibility to infections. Glucocorticoids are known modulators of inflammation, both preventing over‐inflammatory responses and exerting immunosuppressive effects.^4^ Therefore, we hypothesised that chronic exposure to excessive endogenous glucocorticoids elicits these complications through modulation of innate immune responses. In this study, we aimed to assess the effects of chronic hypercortisolism on the innate immune response, specifically focusing on trained immunity.

Nineteen treatment‐naïve CS patients with no comorbidities or medications influencing the immune system and 19 sex‐matched healthy controls were prospectively included in this study (Figure S1). The patients’ baseline characteristics are shown in Table S1. The patients were marginally older than the controls (mean age: 48.8 years vs. 40.7 years), deemed likely not clinically or immunologically relevant. Blood was drawn from patients at diagnosis before the start of cortisol‐lowering medication. Figure S2 shows an overview of the study's inclusions.

First, steroid concentrations were analysed by measuring a panel consisting of cortisol, 11‐deoxycortisol, androstenedione, testosterone, progesterone and 17α‐hydroxy‐progesterone in ethylenediaminetetraacetic acid (EDTA)‐plasma by liquid chromatography‐tandem mass spectrometry, as described elsewhere.^5^ As expected, both cortisol and 11‐deoxycortisol concentrations were higher in CS patients than in healthy controls (Figure 1A). Concentrations of the other steroids were not significantly different in both groups (Figure S3A). Subsequently, subpopulations within peripheral blood mononuclear cells (PBMCs) were analysed. PBMCs from CS patients showed significantly higher percentages of monocytes and neutrophils and lower percentages of lymphocytes than those from healthy controls (Figure 1B). To correlate cell counts to the severity of hypercortisolism, correlations were analysed between plasma cortisol and 11‐deoxycortisol concentrations and PBMC fraction percentages (Figure 1C). Both plasma cortisol and 11‐deoxycortisol showed significant positive and negative correlations to percentages of monocytes and lymphocytes within PBMCs, respectively. The other plasma steroid concentrations were not significantly correlated with PBMC percentages (Figure S3B).

**FIGURE 1:**
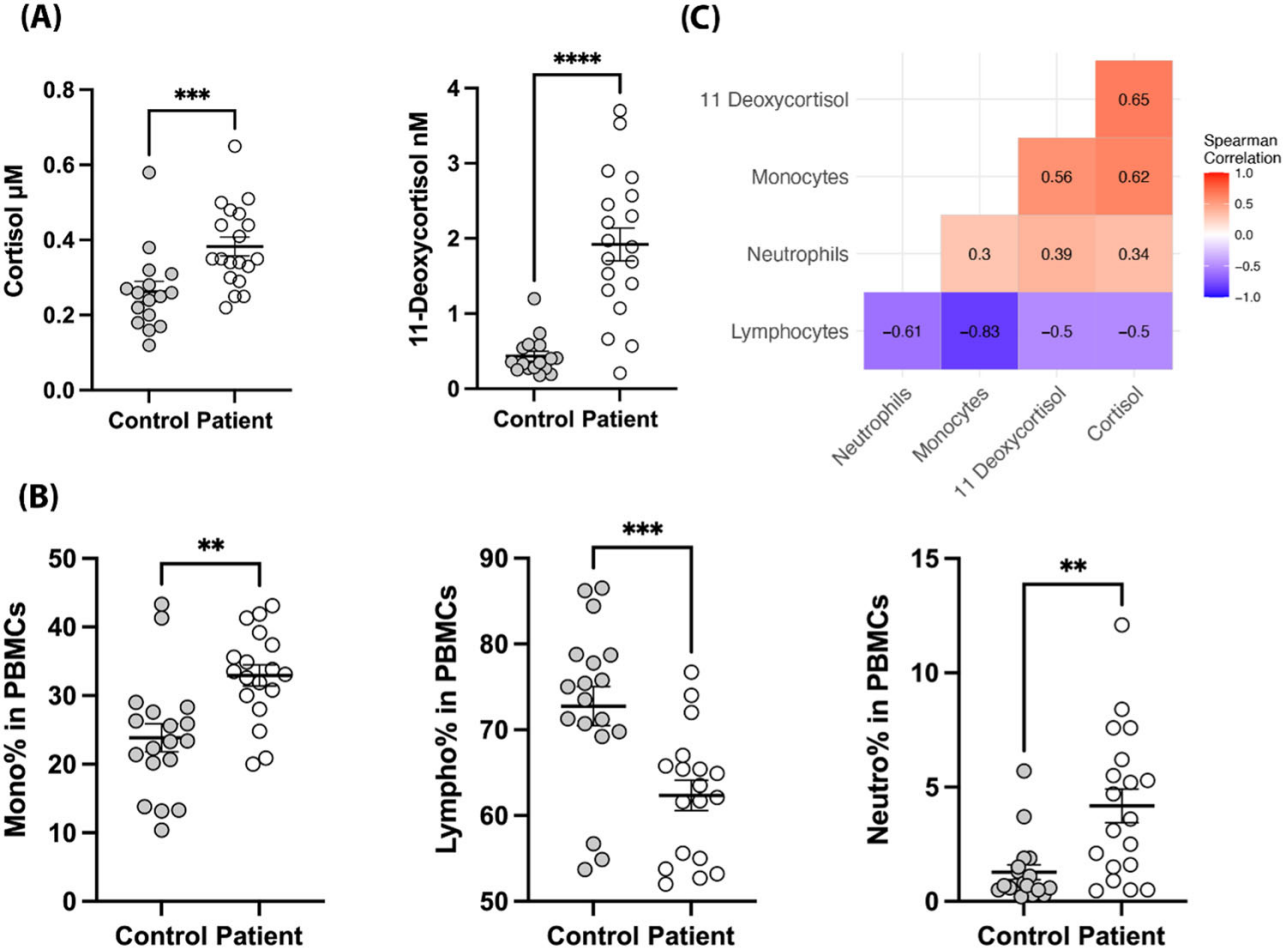
Characteristics of immune cell composition and plasma steroids. (A) Percentages of monocytes, lymphocytes and neutrophils in the peripheral blood mononuclear cell (PBMC) fraction of healthy controls and Cushing's syndrome (CS) patients. Cell counts were measured using a Sysmex XN‐450 automated hematology analyser (Sysmex, Kobe, Japan). (B) Plasma concentrations of cortisol and 11‐deoxycortisol from healthy controls and CS patients. Individuals receiving oral contraceptives were excluded from analysis. (C) Heatmap of correlation coefficients between plasma cortisol and 11‐deoxycortisol and PBMC fraction percentages. Data are presented as mean ± standard error of mean (SEM). ^**^ *p* < .01, ^***^ *p* < .001, ^****^ *p* < .0001, Mann–Whitney *U*‐test. The correlation between cell composition and steroids was assessed using Spearman's correlation.

Next, the inflammatory proteome of CS patients and healthy controls was assessed. Principal component analysis showed two distinct groups (Figure 2A). Analysis revealed 12 inflammation‐related proteins with a higher concentration in CS patients (CCL3, CCL11, CUB domain‐containing protein 1 (CDCP1), EN‐RAGE, fibroblast growth factor 21 (FGF‐21), hepatocyte growth factor (HGF), interleukin (IL)‐6, IL‐8, oncostatin‐M (OSM), protransforming growth factor α (TGF‐α), vascular endothelial growth factor A (VEGFA) and eukaryotic translation initiation factor 4E binding protein 1 (4E‐BP1)), while three proteins were downregulated (CD244, IL‐12B and TWEAK) (Figure 2B,C). This suggests systemic inflammation in CS patients. Plasma cortisol concentrations were only significantly correlated with CD244 (*r* = −.591), whereas plasma 11‐deoxycortisol concentrations were significantly positively correlated with HGF (*r* = .515), CCL11 (*r* = .632) and FGF‐21 (*r* = .613) and negatively correlated with TWEAK (*r* = −.532) and IL‐12b (*r* = −.509).

**FIGURE 2:**
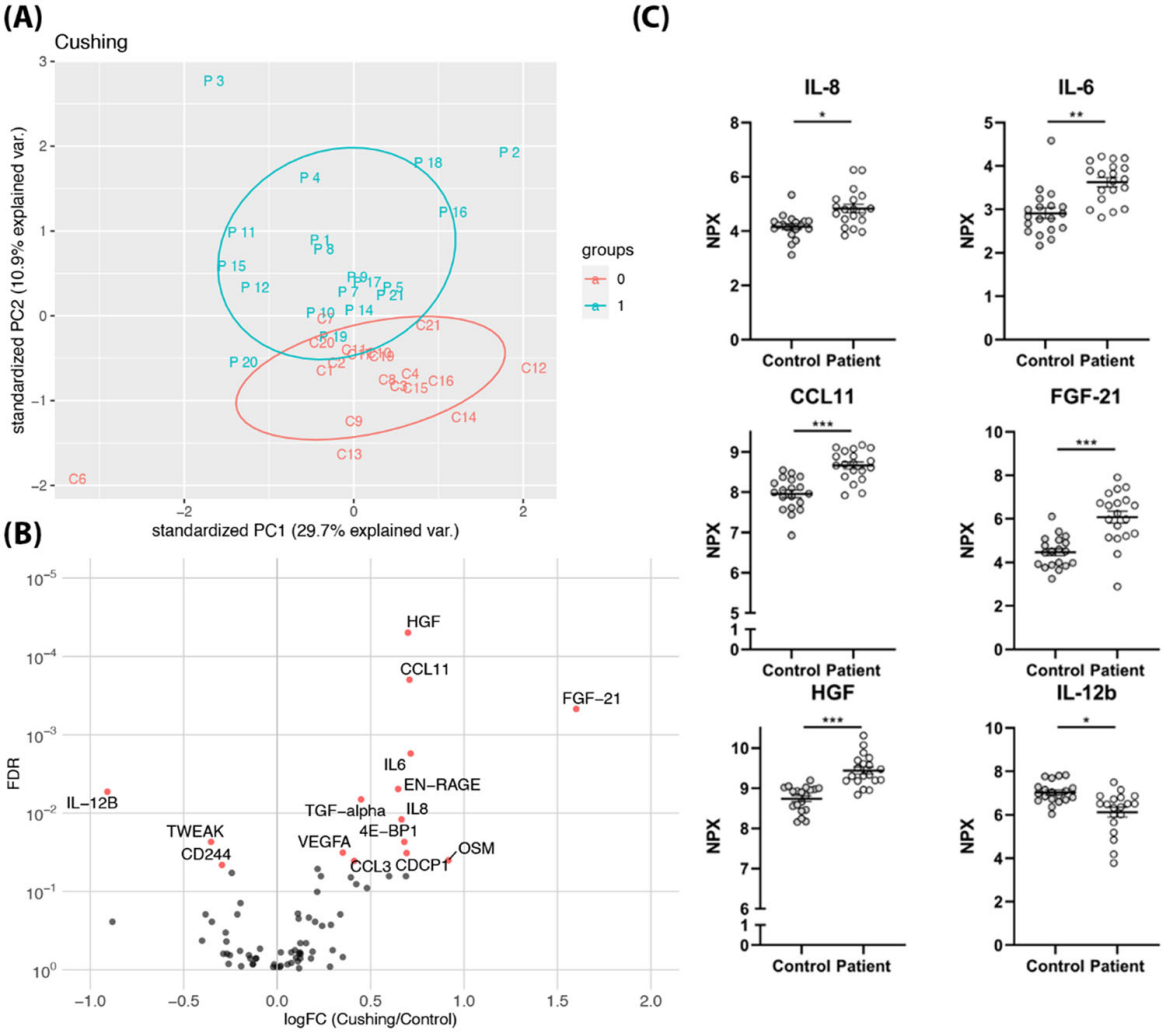
Characteristics of plasma inflammatory proteins. (A) Principal component analysis of 76 inflammation‐related proteins from patients (blue dot, *n* = 19) and healthy controls (red dot, *n* = 19). (B) Differential protein abundance. Volcano plot of log2 fold change differences in protein abundance between Cushing's syndrome (CS) patients and healthy controls. Proteins with false discovery rate (FDR) less than .05 are shown as red dots. (C) Normalised protein expression (NPX) values of IL‐8, IL‐6, CCL11, FGF‐21, HGF and IL‐12b in plasma from healthy controls and CS patients. Data are presented as mean ± standard error of mean (SEM). ^*^ *p* < .05, ^**^ *p* < .01, ^***^ *p* < .001, Mann–Whitney *U*‐test. Analysis was performed using R (version 4.2.2) with R packages ‘ggbiplot’ and ‘ggplot2’. Only proteins for which ≥75% of samples were within the limits of detection were included in the analysis.

To assess cytokine production capacity upon stimulation, PMBCs were stimulated ex vivo with different stimuli for 24 hours (Figure 3A), followed by assessment of cytokine concentrations by ELISA. PBMCs from CS patients produced lower amounts of proinflammatory cytokines tumor necrosis factor (TNF‐α), IL‐1β and IL‐6 upon 24 hours of stimulation than those from healthy controls (Figure 3B). Concentrations of IL‐8, IL‐10 and IL‐1RA did not differ between patients and controls. These results suggest an immunotolerant phenotype in CS patients. Cytokine concentrations were not significantly correlated with plasma concentrations of cortisol and 11‐deoxycortisol. However, glucocorticoid concentrations can be highly variable in CS and one value might not be representative of disease severity.^6^ Moreover, other factors such as variants of glucocorticoid receptor genes can play a role in the sensitivity of immune cells towards hypercortisolism. T‐lymphocyte‐derived cytokines IL‐17, IL‐22 and interferon (IFN)‐γ were measured 7 days after PBMC stimulation. Their concentrations were comparable between CS patients and healthy controls (Figure 3C).

**FIGURE 3:**
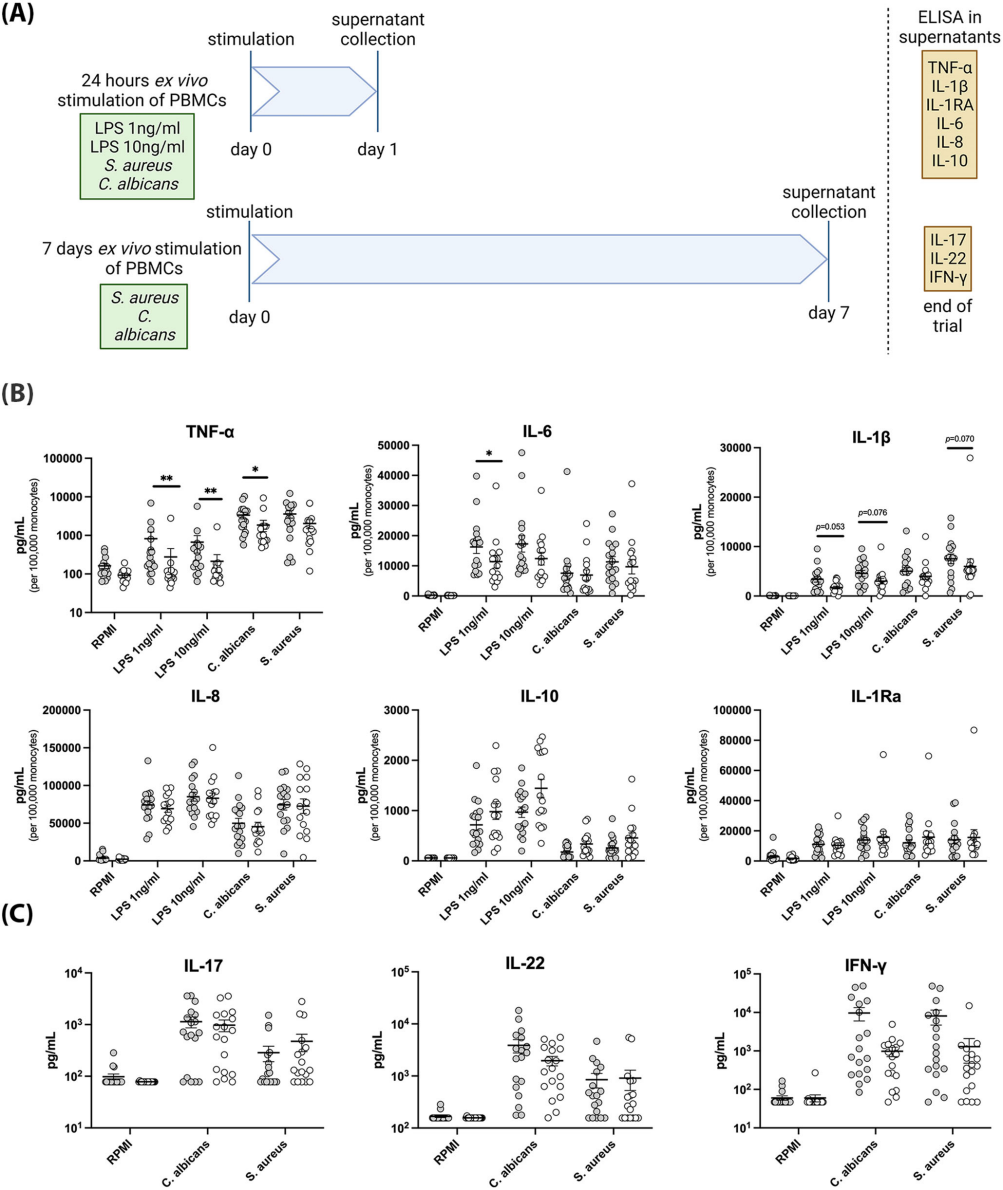
Peripheral blood mononuclear cells (PBMCs) from Cushing's syndrome (CS) patients show an immunocompromised phenotype in ex vivo stimulation experiments. (A) Schematic overview of ex vivo stimulation experiments. Illustration created with BioRender.com (B) Cytokine production after 24 h of stimulation of 500 000 PBMCs with lipopolysaccharide (LPS) 1 ng/mL, LPS 10 ng/mL, *Candida albicans* (ATCC MYA‐3573 UC820) 1 × 10^6^/mL, *Staphylococcus aureus* (Rosenbach ATCC 25923) 1 × 10^6^/mL or Roswell Park Memorial Institute (RPMI) medium as control. Healthy controls are shown as grey dots, CS patients as white dots. Cytokine concentrations are normalised as per 100 000 monocytes. (C) Cytokine production after 7 days of stimulation of 500 000 PBMCs with *C. albicans*, *S. aureus* or RPMI medium as control. Stimulation was performed in medium supplemented with 10% human pooled serum. Data are presented as mean ± standard error of mean (SEM). ^*^ *p* < .05, ^**^ *p* < .01, Mann–Whitney *U*‐test.

Lastly, we investigated the influence of chronic hypercortisolism on the induction of trained immunity by performing an in vitro trained immunity model with monocytes, as described elsewhere^7^ (Figure 4A). Both β‐glucan and *Candida albicans* were able to induce trained immunity in monocytes from CS patients similarly to monocytes from healthy controls. However, interestingly, monocytes from CS patients were unable to mount trained immunity upon exposure to Bacillus Calmette‐Guérin (BCG), in contrast to healthy controls (Figure 4B). The different pathways through which trained immunity is induced in monocytes by different stimuli, either via dectin‐1 for β‐glucan and *C. albicans* or through the NOD2 receptor for BCG, might explain these differences.^8^, ^9^ Remarkably, TNF‐α and IL‐6 concentrations after restimulation with lipopolysaccharide (LPS) of β‐glucan‐primed monocytes showed strong correlations with 24‐h urine free cortisol concentrations in CS patients (Figure 4C).

**FIGURE 4:**
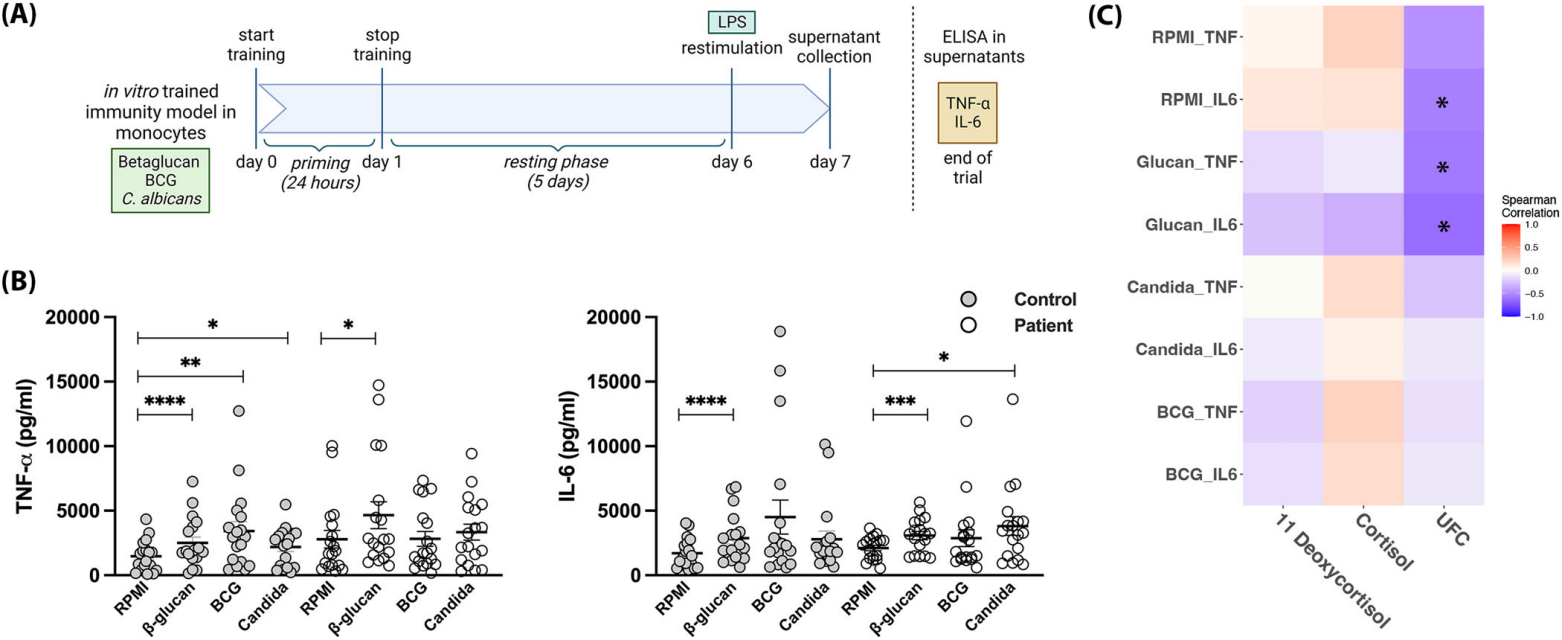
Schematic overview of in vitro trained immunity model in monocytes. Monocytes were isolated from peripheral blood mononuclear cells (PBMCs) using hyperosmotic Percoll gradient isolation (Sigma–Aldrich, St. Louis, MO, USA). Illustration created with BioRender.com. (B) Cytokine production upon 24 hours of restimulation with LPS 10 ng/mL after training. β‐Glucan, BCG and *Candida albicans* trained monocytes versus Roswell Park Memorial Institute (RPMI) culture monocytes were compared for each individual. Data are shown as mean ± standard error of mean (SEM). ^*^ *p* < .05, ^**^ *p* < .01, ^***^ *p* < .001, ^****^ *p* < .0001, nonparametric Wilcoxon matched‐pairs tests. (C) Heatmap of Spearman's correlation coefficients between plasma cortisol and 11‐deoxycortisol and 24 hours urine‐free cortisol (UFC) concentrations and cytokine productions of restimulated trained monocytes. Stars indicate significance of correlation.

In summary, our results indicate that CS patients show paradoxical characteristics of concomitant systemic inflammation and cellular immunosuppression. The former is characterised by a shift towards the myeloid compartment in PBMC composition and increased concentrations of inflammatory proteins in plasma. On the other hand, monocytes from CS patients displayed an exhausted/immunocompromised phenotype, and showed less capacity for ex vivo induction of proinflammatory cytokine production, particularly TNF‐α, IL‐6 and IL‐1β. In addition, monocytes from CS patients displayed defective trained immunity induced by BCG. In CS patients, this might play a role in increased incidences of both cardiovascular complications associated with the systemic inflammatory profile, and infections potentially in the context of a reduced ability to produce cytokines upon stimulation by pathogens. Future studies with larger populations are needed to further elucidate the specific molecular mechanisms behind these immunological alterations and to assess their reversibility after achieving remission of the disease. Focusing on the single‐cell metabolic landscape of tumour cells, cells in the tumour microenvironment and peripheral immune cells, together with hormonal interference in these processes, will be paramount to understand the immune dysregulation in CS.^10^ These findings might be of importance not only for the management of CS patients but also for patients treated chronically with GCs.

## CONFLICT OF INTEREST STATEMENT

The authors declare they have no conflicts of interest.

## Supporting information

Supporting Information

## Data Availability

Data are available on reasonable request from the corresponding author.
