# Supplementary material for: Concomitant systemic inflammation and cellular immunosuppression in patients with Cushing's syndrome
Source: Clin Transl Med. 2023 Jul 13;13(7):e1314. doi: 10.1002/ctm2.1314 (PMC10345458; doi:10.1002/ctm2.1314)
Supplement: Supplementary file 1 — Supporting Information [file CTM2-13-e1314-s001.docx]

**Supplementary material**

Concomitant systemic inflammation and cellular immunosuppression in patients with Cushing’s syndrome

**Supplementary Table**

| *Baseline characteristics (n=19)* | |  |
| --- | --- | --- |
| Cause of Cushing’s syndrome (CD/ACS) | | 8/11 |
| Sex (male/female) | | 4/15 |
| BMI at diagnosis (kg/m^2^) | | 29.01 ±3.18 |
| Age at diagnosis (years) | | 48.8 ± 11.3 |
| Duration of symptoms at diagnosis (months) | | 60.61 ± 47.90 |
| Cortisol after 1-mg DST at diagnosis (umol/L) | | 0.36 ± 0.12 |
| 24-hours UFC at diagnosis (nmol) | | 320 ± 247 |
| 24-hours UFC at diagnosis (times upper limit of normal) | | 2.37 ± 1.82 |
| Midnight salivary cortisol at diagnosis (nmol/L) | | 6.06 ± 4.81 |
|  | |  |
| *Comorbidities (frequencies)* | | *n* |
| Diabetes mellitus | | 2 |
| HbA1c (mmol/mol) | 42.6 ±9.08 | |
| Hypertension | | 12 |
| Dyslipidemia | | 8 |
| Renal insufficiency | | 1 |
| Obesity | | 8 |
| Heart failure | | 1 |
| Venous thrombo-embolism | | 1 |
|  | |  |

Supplemental Table 1

Baseline characteristics from all included CS patients. CD: Cushing’s disease, ACS: adrenal Cushing’s syndrome, BMI: body mass index, 1-mg DST: 1 milligram overnight dexamethasone suppression test, UFC: urine free cortisol. Data are presented as mean ±standard deviation (SD).


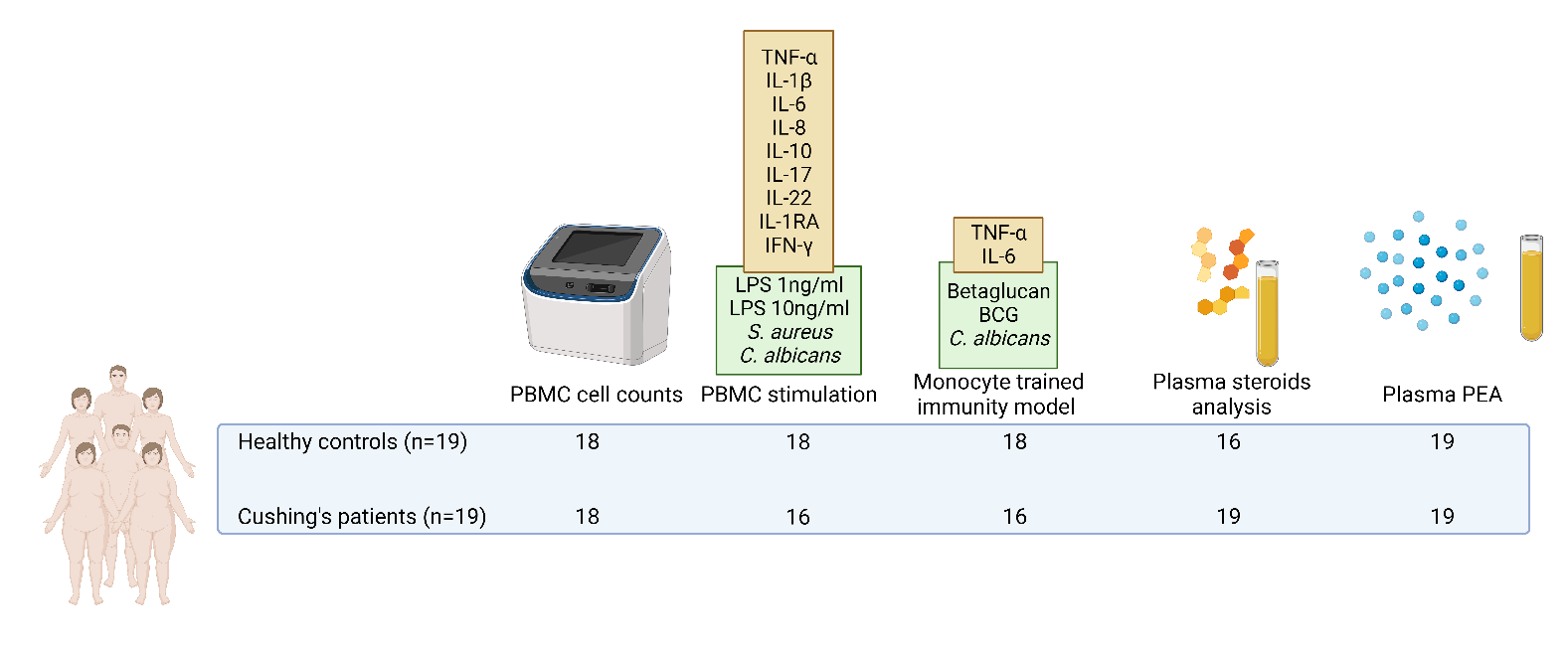
**Supplementary Figures**

Supplemental Figure S1

Overview of study procedures and numbers of participants included in the different analyses. Created with BioRender.com


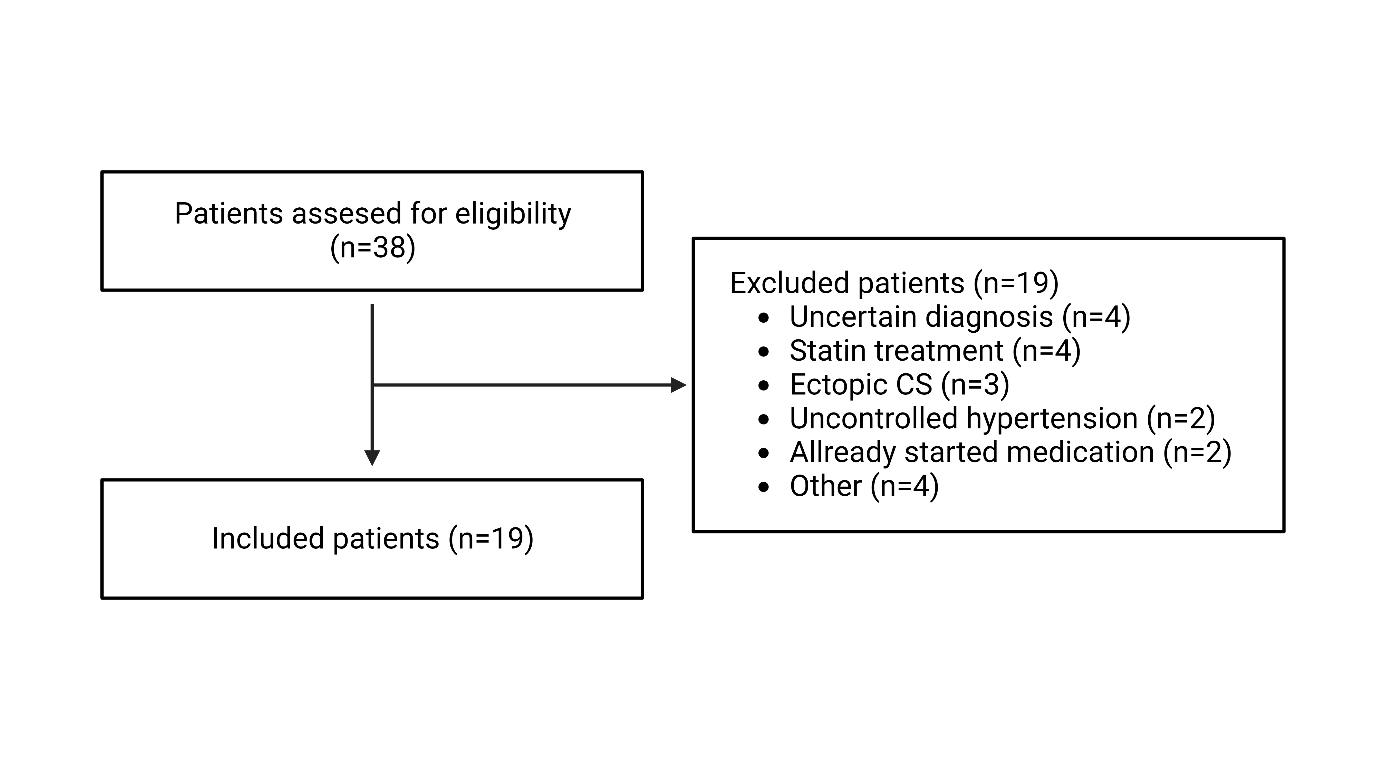


Supplemental Figure S2

Flowchart describing the assessment for eligibility of patients with Cushing’s syndrome and numbers of included and excluded patients and reasons for exclusion.


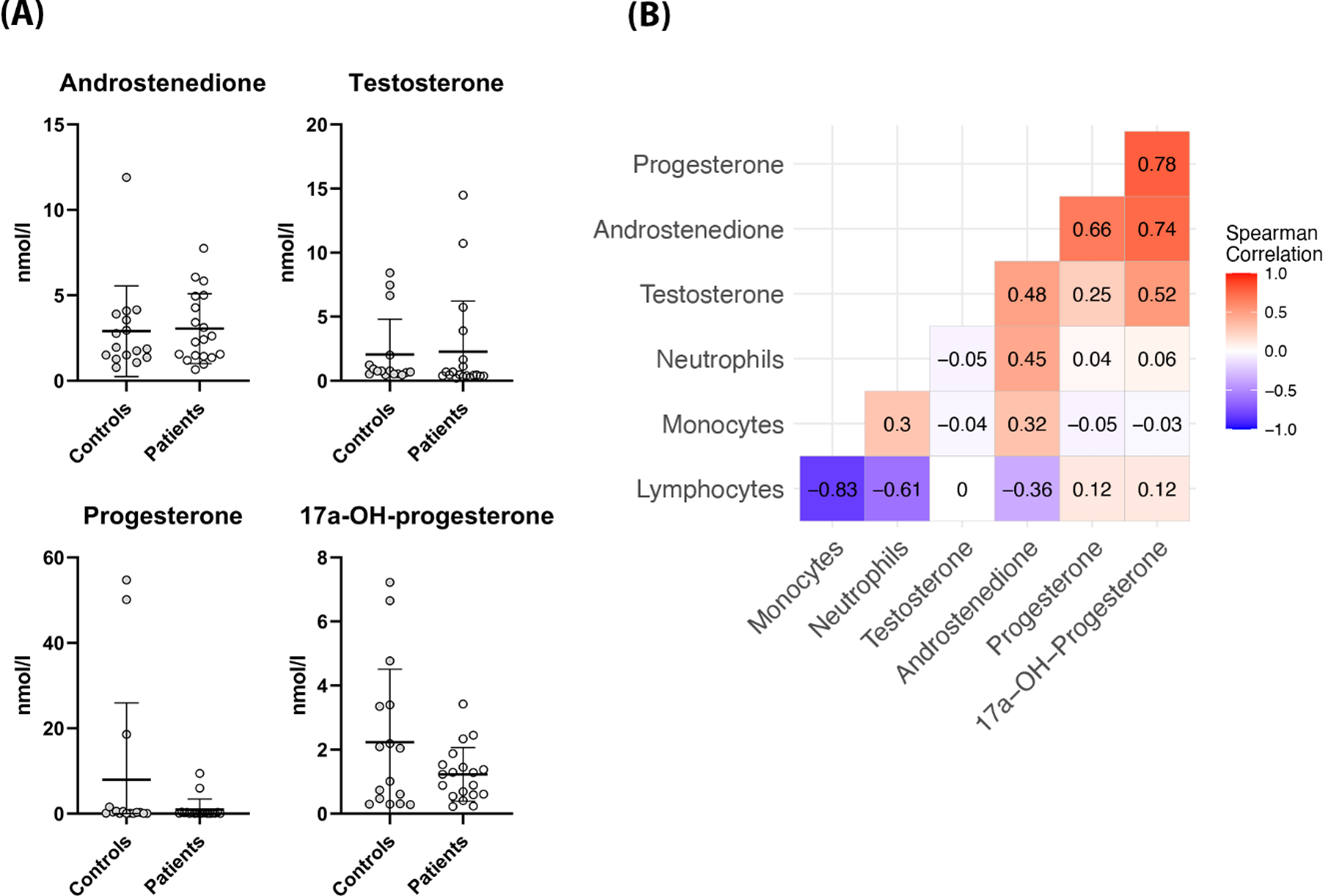


Supplemental Figure S3

No significant correlations between PBMC fractions and other steroids in plasma. (A) Plasma concentrations of androstenedione, testosterone, progesterone and 17a-hydroxyprogesterone in healthy controls (n=16) and CS patients (n=19), individuals receiving oral contraceptives were excluded from analysis. Data are presented as mean ± Standard error of mean (SEM). (B) Spearman correlation coefficients for PBMC fraction percentages and plasma concentrations of androstenedione, testosterone, progesterone and 17a-hydroxyprogesterone.

**Supplementary Materials and Methods**

1. **Subjects and study procedures**

All consecutive adult patients (18 years and older) diagnosed with CS and presenting at the Radboud University Medical Center in Nijmegen, the Netherlands, between August 2020 and March 2022 who fulfilled the inclusion criteria were invited to participate in the study. The inclusion criteria were biochemically confirmed CS according to the available guidelines and not started on cortisol lowering medication (1). Exclusion criteria were: inflammatory comorbidities, active malignancies, use of statins, use of systemic immunosuppressive medication, inadequately treated hypertension (defined as systolic blood pressure ≥160 mmHg or diastolic blood pressure ≥100 mmHg), poorly controlled diabetes mellitus (defined as HbA1c concentration of >69 mmol/mol for >1 year), recent ischemic cardiovascular disease (<1 year) and/or a self-reported alcohol intake of >21 units per week.

The study was approved by the local institutional review board (dossier number 2020-6440). Procedures were conducted according to the principles of the Declaration of Helsinki. Informed consent was obtained from all participants before study procedures.

Before starting cortisol lowering medication, blood was obtained from the CS patients via venipuncture. On the same day, blood was obtained from a sex matched healthy donor. The following analyses were performed: immune cell count analysis, plasma hormonal analysis, proximity extension assay (PEA), *ex vivo* peripheral blood mononuclear cell (PBMC) stimulation, *in vitro* monocyte trained immunity model and enzyme-linked immunosorbent assay (ELISA).

Clinical data including age, height, weight, duration of symptoms, cause of CS and biochemical results including 24-hours urine free cortisol levels (UFC) were retrieved from patient files. No UFC levels were available for healthy controls.

1. **Materials**

Blood was drawn using EDTA tubes. To minimize the effect of diurnal rhythm, blood was drawn in patients and sex-matched controls within 1 hour of each other. All experiments were performed in Roswell Park Memorial Institute (RPMI) 1640 Dutch Modified (Gibco, Thermo Scientific) supplemented with gentamycin 50 ug/ml, pyruvate 1mM and glutamax 2mM. β-glucan (β-1,3-(D)glucan) was kindly provided by Professor David Williams (College of Medicine, Johnson City, TN, USA). BCG SSI, Danish strain 1331, was provided by AJVaccines, Copenhagen, Denmark. *E. Coli* lipopolysaccharide (LPS; serotype O55:B5; Sigma-Aldrich, St. Louis, MO, USA) was repurified as previously described (2). *Candida albicans* (*C. albicans*) ATCC MYA-3573 (UC 820) and *Staphylococcus aureus* (*S. aureus*) Rosenbach ATCC 25923 were grown overnight at 37°C in Sabouraud and Brain Heart Infusion broth, respectively. Microorganisms were harvested by centrifugation, washed twice in PBS, and resuspended. *C. albicans* yeasts were heat-killed for 30 minutes at 95°C.

1. **PBMCs cellularity**

Cell counts of subpopulations within PBMC fraction were obtained using a Sysmex XN-450 automated hematology analyzer (Sysmex Corporation, Kobe, Japan).

1. **Plasma hormonal assays**

Separated plasma from EDTA tubes was stored at -80 °C until analysis. A panel consisting of cortisol, 11-deoxycortisol, androstenedione, testosterone, progesterone and 17α-hydroxy-progesterone was measured in plasma by liquid chromatography-tandem mass spectrometry (LCMSMS) as described elsewhere (3). Sample preparation was performed by protein precipitation and subsequent solid phase extraction.

Because estrogens upregulate the concentration of cortisol-binding globulin and that of total cortisol, healthy controls that were using oral contraceptives at the time of blood drawing (n=3) were excluded from plasma steroids analyses (4).

1. **Proximity Extension Analysis (PEA)**

Proteomics in plasma from patients and controls was analyzed using a proteomic multiplex assay (Olink, Uppsala, Sweden), specifically the Target 96 Inflammation panel. This analysis simultaneously quantifies 92 inflammation related proteins. For each protein, two separate antibodies are connected to one oligonucleotide each. Then, the 3’ ends of the oligonucleotides hybridize, priming a DNA polymerization reaction that forms protein-specific reporter DNA sequences for each detected protein. These DNA strands are quantified using quantitative real-time polymerase chain reactions (5). All samples were analyzed on the same plate together with internal and external controls.

1. **Isolation of PBMCs and *ex vivo* PBMC stimulation**

Peripheral blood mononuclear cells (PBMCs) were isolated using Ficoll-Paque PLUS (GE Healthcare, Diegem, Belgium). Stimulation experiments were performed in round-bottom 96-well plates. Per well, 5 x 10^5^ PBMCs were stimulated in duplicate for either 24 hours with LPS 1ng/ml, LPS 10ng/ml, *S. aureus* 1x10^6^/ml, *C. albicans* 1x10^6^/ml or RPMI medium as control, or 7 days with *S. aureus* 1x10^6^/ml, *C. albicans* 1x10^6^/ml or RPMI medium as control. The 24 hours and 7 days stimulation experiments were used for assessing the monocyte and T-cell responsiveness to stimuli respectively. Earlier studies have shown that production of T-cell derived cytokines upon PBMC stimulation with *C. albicans* or *S. aureus* is considerably higher than upon stimulation with LPS (6, 7). Therefore we have chosen to use *C. albicans* and *S. aureus* as stimuli in the 7 days stimulation experiment. After 24 hours or 7 days of incubation at 37°C, 5% CO_2_, supernatants were collected and stored at -80°C until measurement. The 7 days stimulation was performed in medium supplemented with 10% human pooled serum.

1. ***In vitro* trained immunity model in monocytes**

The monocyte fraction of PBMCs was enriched using hyperosmotic Percoll gradient isolation (Sigma-Aldrich, St. Louis, MO, US) (8). Training of adherent monocytes was performed in flat-bottom 96-well plates as described previously (9). Per well, 1.5 x 10^5^ adherent monocytes were stimulated for 24 hours with β-glucan 1 ug/ml, BCG 5 ug/ml, *C. albicans* 1x0^5^/ml or RPMI medium as control. After 24 hours of training, cells were washed and fresh medium supplemented with 10% human pooled serum was added. After 5 days of resting, cells were restimulated with LPS 10 ng/ml or RPMI medium as control. After 24 hours of restimulation, supernatants were collected and stored at -80 °C until measurement.

1. **Cytokine concentration measurements**

Cytokine production after (re)stimulation was determined in supernatants with commercial ELISA kits for IL-1β, IL-6, IL-8, IL-10, IL-17, IL-22, IL-1 receptor antagonist (IL-1RA), TNF-α and interferon-γ (IFN-γ) (R&D Systems, Minneapolis, MN, USA) in accordance with the instructions of the manufacturer. Samples from one patient and the sex matched control were measured in the same ELISA plate. IL-1β, IL-6, IL-8, IL-10, IL-1RA and TNF-α were measured in supernatants from 24 hour-stimulated PMBCs and were regarded as monocyte-derived. IL-17, IL-22 and IFN-γ were measured in supernatants from 7 days stimulated PBMCs and, while lymphocyte-derived, were regarded as the result of the interplay between lymphocytes and monocytes.

1. **Statistical analysis**

Data were analyzed using SPSS (version 25.0; SPSS Inc., Chicago, IL, USA) and GraphPad Prism (version 8.02; GraphPad Software, La Jolla, CA, USA). Normal distribution was tested using Shapiro-Wilk test. Categorical variables were compared using Chi-squared test. Continuous variables were compared using Mann-Whitney U test for unpaired data and Wilcoxon signed-rank test for paired data. In order to correctly assess the cytokine production capacity, the level of monocyte-derived cytokines in the 24-hours stimulation assay was normalized by dividing the cytokine concentration (in pg/mL) by cell count (as per 100,000 cells).

PEA results were analyzed and visualized using R (version 4.2.2), with R packages “ggbiplot” and “ggplot2”. Only proteins of whom ≥75 of samples was above limit of detection were included in the analysis. Normalized Protein Expression (NPX) values were expressed on a log2 scale and were linearized for fold-change calculations. Protein concentrations of CS patients and healthy controls were compared using Mann-Whitney U test. To adjust for multiple testing, false discovery rate (FDR) correction was performed.

Correlations were analyzed using the Spearman’s test. Correlations between plasma cortisol and 11-deoxycortisol and inflammatory proteins were analyzed in the significantly up- or downregulated proteins and were corrected for multiple testing by use of the Bonferroni correction.

All statistical analyses were performed two-tailed. *P* < 0.05 was considered statistically significant.

**References**

1. Nieman LK, Biller BM, Findling JW, et al. The diagnosis of Cushing's syndrome: an endocrine society clinical practice guideline. The Journal of Clinical Endocrinology & Metabolism. 2008;93(5):1526-40.

2. Hirschfeld M, Ma Y, Weis JH, et al. Cutting edge: repurification of lipopolysaccharide eliminates signaling through both human and murine toll-like receptor 2. The Journal of Immunology. 2000;165(2):618-22.

3. Aguirre-Gamboa R, Joosten I, Urbano PC, et al. Differential effects of environmental and genetic factors on T and B cell immune traits. Cell reports. 2016;17(9):2474-87.

4. Qureshi AC, Bahri A, Breen LA, et al. The influence of the route of oestrogen administration on serum levels of cortisol‐binding globulin and total cortisol. Clinical endocrinology. 2007;66(5):632-5.

5. Assarsson E, Lundberg M, Holmquist G, et al. Homogenous 96-plex PEA immunoassay exhibiting high sensitivity, specificity, and excellent scalability. PloS one. 2014;9(4):e95192.

6. Bruno M, Davidson L, Koenen HJ, et al. Immunological Effects of Anti‒IL-17/12/23 Therapy in Patients with Psoriasis Complicated by Candida Infections. Journal of Investigative Dermatology. 2022;142(11):2929-39. e8.

7. Rosati D, Bruno M, Jaeger M, et al. An exaggerated monocyte-derived cytokine response to Candida hyphae in patients with recurrent vulvovaginal candidiasis. The Journal of infectious diseases. 2022;225(10):1796-806.

8. Repnik U, Knezevic M, Jeras M. Simple and cost-effective isolation of monocytes from buffy coats. Journal of immunological methods. 2003;278(1-2):283-92.

9. Domínguez-Andrés J, Arts RJ, Bekkering S, et al. In vitro induction of trained immunity in adherent human monocytes. STAR protocols. 2021;2(1):100365.
